# Symptomatic femoral head necrosis in patients with rheumatoid arthritis: A retrospective case‐control study

**DOI:** 10.1002/iid3.633

**Published:** 2022-05-11

**Authors:** Qijiao Wei, Qing Yan, Diantian Lin, Fei Gao, He Lin, Zhihan Chen

**Affiliations:** ^1^ Department of Rheumatology Fujian Provincial Hospital Fuzhou China; ^2^ Fujian Medical University Provincial Clinical Medical College Fuzhou China

**Keywords:** antiphospholipid antibody, antiphospholipid antibody score, osteonecrosis of the femoral head, rheumatoid arthritis, risk factors

## Abstract

**Objective:**

We conducted a retrospective case–control study to investigate the risk factors for osteonecrosis of the femoral head (ONFH) in rheumatoid arthritis (RA) patients.

**Methods:**

The clinical data of patients diagnosed with RA at Fujian Provincial Hospital from January 2013 to December 2020 were retrospectively collected and evaluated. Twenty‐two patients with ONFH were identified. Eighty‐eight age‐, sex‐, and disease duration‐matched RA patients without symptomatic ONFH were randomly selected as controls in a ratio of 1:4. Logistic regression analysis was used to analyze the risk factors.

**Results:**

The anticardiolipin (ACL)‐immunoglobulin G (IgG) concentration, clinical disease activity index, simplified disease activity index, incidence of hyperlipidemia in the case group were higher than those in the control group. Multivariate logistic regression analysis did not find the independent risk factor in ONFH patients with RA.

**Conclusion:**

The pathogenesis of ONFH in RA is related to many factors such as ACL IgG, disease activity index, and hyperlipidemia. While, we went to great lengths to explore the relationship between antiphospholipid antibodies and ONFH, but it plays a very small role.

## Graphical abstract

This study analyzed the risk factors for osteonecrosis of the femoral head (ONFH) in patients with rheumatoid arthritis (RA) in a single center over a period of 8 years. The longest follow‐up time was 20.5 years, which was in a real‐world study. It is the first time to explore the relationship between antiphospholipid antibody and ONFH in RA. While antiphospholipid antibody is not the key risk factor for ONFH.

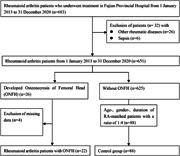

## 1 INTRODUCTION

Rheumatoid arthritis (RA) is a systemic autoimmune disease. Its main clinical manifestations are joint synovitis and chronic polyarthritis.^1^ Severe RA can destroy the articular cartilage, bones, and joints, and even cause bone degeneration and necrosis. It has been reported that the incidence of RA with osteonecrosis of the femoral head (ONFH) is approximately 5.0%–6.0%, which is significantly higher than that in the normal population.^2^ This suggests the presence of risk factors specific to RA. At present, there are few studies on the risk factors for ONFH in patients with RA. Among patients with systemic autoimmune diseases, abnormal lipid metabolism, hypercoagulability, oxidative stress, and vascular endothelial dysfunction have been hypothesized to be involved in the development of ONFH. Some studies have reported that systemic lupus erythematosus (SLE) patients demonstrate an association between antiphospholipid antibodies (APLs) and ONFH. Therefore, a high antiphospholipid antibody score (APL‐S) may be an important risk factor for ONFH development. Do APLs also have an impact on RA patients? This study reviewed the clinical data and laboratory indicators of 110 RA patients in a single center and analyzed the risk factors, especially APLs, to guide clinicians.

## 2 MATERIALS AND METHODS

### 2.1 Participants

**Figure 1:**
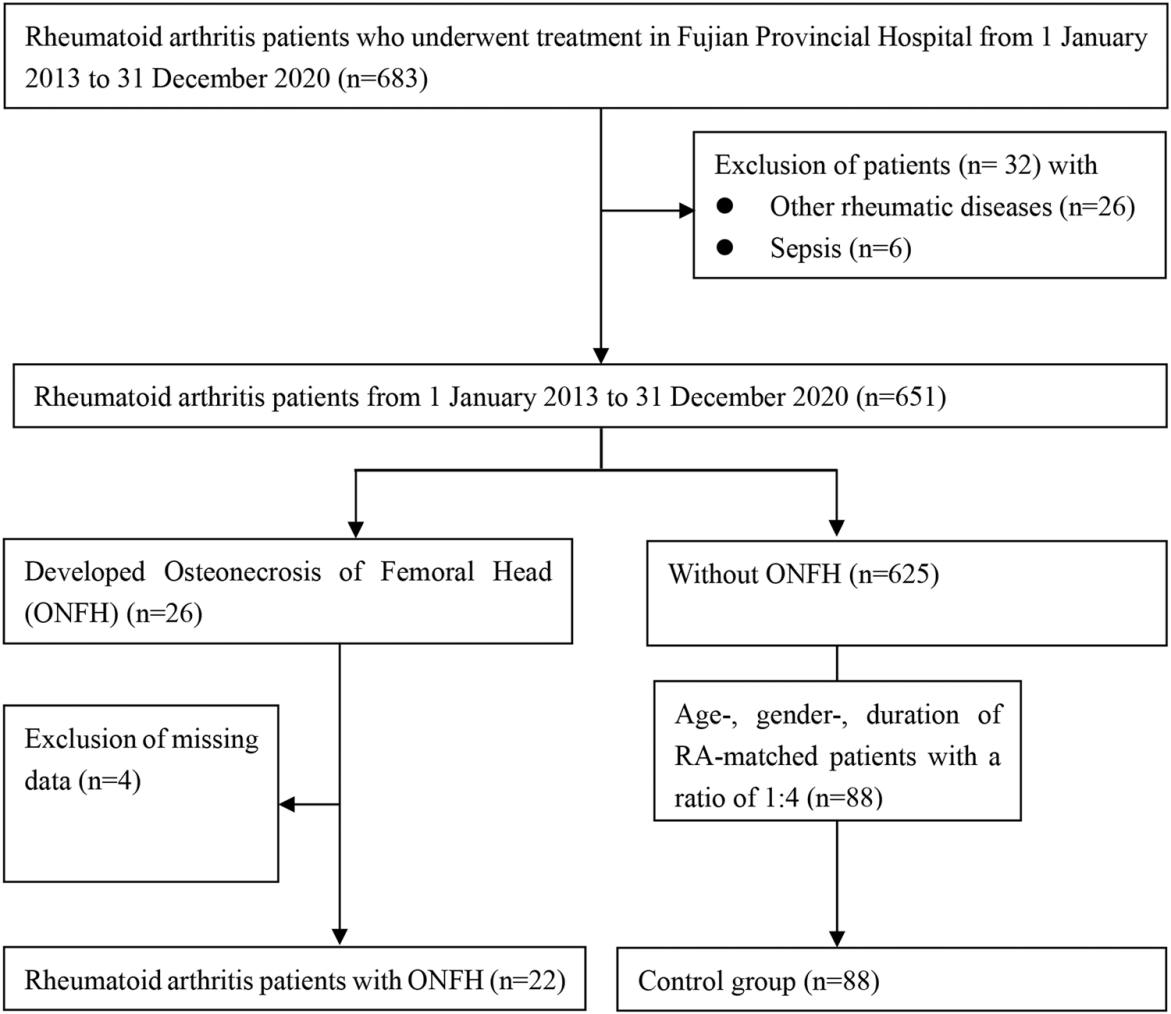
Study flow diagram.

A total of 683 patients with RA were diagnosed between January 2013 and December 2020 in the Department of Rheumatology. Twenty‐two patients with ONFH reported pain in the hip or groin or with shortening of the legs or difficulty in walking. They were confirmed by magnetic resonance imaging (MRI) and/or X‐ray examination. Eighty‐eight age‐, sex‐, and disease duration‐matched RA patients without symptomatic ONFH were randomly selected as controls in a ratio of 1:4 (Figure 1). All patients met the 2010 ACR/EULAR classification criteria for RA.^3^ Patients with other rheumatic diseases, tumors, and incomplete medical records at baseline were excluded from this case–control study.

### 2.2 Clinical data

The demographic, clinical, and laboratory characteristic data of the patients included in this study were collected retrospectively from outpatient and inpatient medical records. The clinical data included general parameters, such as sex, age of onset, duration of RA before ONFH, body mass index (BMI), alcohol consumption, and smoking status. The comorbidities included diabetes mellitus, hyperlipidemia, hypertension, and osteoporosis. The laboratory measurements included platelet count (PLT), albumin (Alb), immunoglobulin G (IgG), anticardiolipin (ACL) and anti‐β2GP1 antibodies, fibrinogen, activated partial thromboplastin time (APTT), d‐dimers, rheumatoid factor (RF), anti‐cyclic peptide containing citrulline (anti‐CCP), C‐reactive protein (CRP), erythrocyte sedimentation rate (ESR), disease activity score (DAS28 score), clinical disease activity index/simplified disease activity index (CDAI/SDAI) at the time of ONFH, and treatment before ONFH (including whether the patients were using glucocorticoids [GC] or not, the initial dose of the GC, the maximal dose of the GC, hydroxychloroquine, or biologic therapy at baseline, and whether the patients were on regular medications or not).

### 2.3 Definitions

According to the "China Chronic Diseases and Risk Factors Monitoring Report (2007)",^4^ heavy drinking is defined as a daily alcohol intake of >100 g. Hyperlipidemia refers to hypercholesterolemia and/or hypertriglyceridemia. Plasma total cholesterol concentration >5.2 mmol/L is defined as hypercholesterolemia, and plasma triglyceride concentration >1.7 mmol/L is defined as hypertriglyceridemia.^5^ Fibrinogen levels >3.5 g/L are considered to be elevated.^6^ An elevated d‐dimer level is defined as a concentration >0.50 mg/L in patients aged ≤50 years and a concentration >age × 0.01 mg/L in patients aged >50 years. Thromboembolic events were defined as reported by Chen et al.^7^ APLs were assayed according to the standard instructions of the ACL IgG detection kit (EA 1621‐9601 G), IgM detection kit (EA 1621‐9601 M), and anti‐β2GPI IgA/IgG/IgM detection kit (EA 1632‐9601 P) from EUROIMMUN Medical Diagnostics (China) Co., Ltd. The normal ranges were as follows: ACL IgG, >12.0 RU/ml; ACL IgM, 12.0 RU/ml; and Anti‐β2GPI IgA/IgG/IgM, >20 RU/ml. The 99th percentile of the levels in 200 healthy controls was used as the cutoff level for positivity. Any one of these three antibody concentrations exceeding the normal value was considered positive. APL‐S is a quantitative marker of APL and the development of thrombotic events in autoimmune diseases. The patients were divided into a thromboembolic event group and a nonthromboembolic event group. We defined thromboembolic events as stroke, transient ischemic attack, lacunar infarction, myocardial infarction, coronary atherosclerotic heart disease, and superficial thrombophlebitis documented by Doppler ultrasound. For each APL item, we calculated the mean antibody levels in patients with RA and used them as the second cutoff. We calculated the relative risk of thrombosis for each APL item. The final score was calculated using the following formula: APL‐S = 5×exp[(OR‐5)/4]. A partial APL‐S score for each patient was calculated by adding the scores of APTT, IgG/IgM ACL, LA, and anti‐β2GP1. A simplified partial APL‐S was developed using only APTT, IgG/IgM ACL, and anti‐β2GP1.^7^, ^8^

### 2.4 Patient and public involvement

We conducted a retrospective case–control study. And we collected the patient's past medical records. So, patients' priorities, experience, and preferences are not available. In this study, I collected and analyzed clinical data. Patients did not involve in the recruitment to and conduct of the study. We shared our findings with patients by phone or email.

### 2.5 Ethics approval

This study was approved by the institutional review board of the Fujian Provincial Hospital (Ethics approval number: K2021‐01‐032).

### 2.6 Statistical methods

The data were expressed as mean ± standard deviation ($\bar{x}$±s) for normally distributed continuous variables or as median (Q1, Q3) for continuous variables that did not conform to the normal distribution. Ratios were expressed as percentages (%). SPSS (version 25.0; SPSS Inc.) was used to compare the differences between the two groups using the *t* test or Mann–Whitney *U* test. Ratios were assessed using the *χ* ^2^ test. Logistic regression analysis was used to analyze the risk factors. Univariate analysis was performed to determine the variables associated with ONFH. Multivariate logistic stepwise regression was performed with all independent variables with *p* < 0.05. A stepwise forward method was used for variable selection. Statistical significance was set at *p* < 0.05. Not all patients underwent APL testing, so analysis of existing data may introduce bias.

## 3 RESULTS

### 3.1 Clinical features of the 22 RA patients with ONFH

In total, 683 patients with RA were diagnosed between January 2013 and December 2020 in the Department of Rheumatology. Twenty‐six patients with ONFH were confirmed using X‐ray or MRI examinations. The incidence rate of ONFH was 3.8%, and 22 patients with complete medical records were included in this study. Among the 22 patients, 12 were women and 10 were men. The male to female ratio was 1:1.2, the average age of onset was 55.7 ± 13.0 years, and the average duration of RA before ONFH was 10 (4, 20.5) years. Nine patients had bilateral femoral head involvement, six patients had left‐sided ONFH, and seven patients had right‐sided ONFH. One patient had bilateral humeral necrosis (Table 1).

**Table 1:** Clinical features of 22 rheumatoid arthritis (RA) patients with osteonecrosis of the femoral head (ONFH)

	Gender	Age	Duration of RA Before ONFH (year)	Imaging method	Staging of ONFH	CDAI	SDAI	DAS28 at the time of ONFH	Location of ONFH		Other osteonecrosis location	
									Bilateral	Unilateral	
1	M	58	30	MRI	IV	13	12	2.4	+		‐	
2	F	61	20	MRI	IV	136	57	7.2	+		‐	
3	M	64	5	MRI	IV	158	58	7.5		+left	‐	
4	F	49	17	MRI	III‐IV	35	24	5.6		+left	‐	
5	F	68	23	X	3	92	44	6.7		+right	‐	
6	F	46	10	MRI	right II、left III‐IV	82	53	7.0	+		‐	
7	M	56	0.3	MRI	III	114	50	6.9		+right	‐	
8	M	53	3	MRI	IV	114	46	6.9		+left	‐	
9	M	27	3.5	MRI	II	5	4	1.6	+		‐	
10	M	52	6	MRI	II	54	26	4.8		+right	‐	
11	M	46	4	MRI	II‐III	146	56	7.6		+right	‐	
12	F	55	5	X	3	50	47	6.1	+		‐	
13	M	48	10	MRI	I	127	48	6.9	+		‐	
14	F	68	17	MRI	I	72	25	5.2		+left	‐	
15	F	32	7	MRI	I	14	4	3.7		+right	‐	
16	F	54	22	MRI	III	159	42	6.8	+		‐	
17	M	55	24	MRI	II	89	57	6.2	+		‐	
18	F	80	0.3	MRI	III	137	20	5.1		right	Bilateral humerus	
19	F	77	30	MRI	III	69	50	6.9		left	‐	
20	M	55	16	MRI	III‐IV	93	46	6.5		left	‐	
21	F	74	4	MRI	III‐IV	80	52	7.0		right	‐	
22	F	48	20	MRI	II	67	53	7.0	+		‐	

Abbreviations: CDAI, clinical disease activity index; MRI, magnetic resonance imaging; SDAI, simplified disease activity index.

### 3.2 Demographic data and clinical characteristics at baseline

No statistical differences were found between the two groups in terms of sex, age, duration of disease, BMI, smoking, and history of heavy drinking. The incidence of hyperlipidemia in the case group was significantly higher than that in the control group (50.0% vs. 22.7%, *p* = 0.02). The clinical manifestations of extra‐articular disease, including rheumatoid nodules or vasculitis, pulmonary interstitial disease, kidney disease, Felty syndrome, and eye disease were not different. The SDAI and DAS28 (ESR) of the case group were significantly higher than those of the control group (*p* < 0.05) (Table 2).

**Table 2:** Clinical features of patients with or without ONFH

	ONFH group (*n* = 22)	Control group (*n* = 88)	*p* value
General data			
Gender (F/M)	12/10	48/40	1.00
Age (years)	55.7 ± 13.0	57.9 ± 11.6	0.45
Disease duration (years)	12.6 ± 2.0	11.2 ± 1.0	0.52
BMI (kg/m^2^)	22.0 ± 3.3	21.7 ± 4.2	0.75
Smoking (yes/no)	3/19	20/68	0.56
Heavy drinking (yes/no)	1/21	7/81	0.58
Comorbidities			
Diabetes mellitus (yes/no)	3/19	8/80	0.46
Hyperlipidemia (yes/no)	11/11	20/68	0.02
Hypertension (yes/no)	5/17	12/76	0.33
Osteoporosis (yes/no)	15/7	44/44	0.16
Cardiovascular event			
Atherosclerosis (yes/no)	7/15	30/58	1.00
Coronary atherosclerotic heart disease (yes/no)	1/21	3/85	1.00
Articular manifestations			
SDAI	39.7 ± 17.6	26.7 ± 15.5	0.01
CDAI	72.0 ± 51.9	87.5 ± 47.4	0.22
DAS 28 (ESR)	6.0 ± 1.6	5.3 ± 1.3	0.03
Extra‐articular manifestations			
Rheumatoid nodules or vasculitis (yes/no)	4/18	15/73	0.90
RA‐ILD (yes/no)	1/21	2/86	0.49
Nephritis (yes/no)	0/22	2/86	1.00
Felty syndrome (yes/no)	0/22	0/88	1.00
Eye disease (yes/no)	1/21	0/88	0.20

Abbreviation: CDAI, clinical disease activity index; ONFH, osteonecrosis of the femoral head; SDAI, simplified disease activity index.

### 3.3 Partial APL‐S in RA patients

We calculated the relative risk of thrombosis for each APLs. (Table 3). For each patient, we calculated the partial APL‐S. Among the 35 patients with RA whose APL was detected, the partial APL‐S ranged from 0 to 15.17. While, the partial APL‐S has no significant difference between the case group and the control group [2.97 (2.06. 5.49 vs. 2.06 [0, 4.82], *p* = 0.35).

**Table 3:** Relative risk of thrombosis for each APL test

Test	Cutoff	Sensitivity	Specificity	OR (95% CI)	APL‐score
APTT	＞32.5 s	28.6%	76.6%	1.31 (0.55–3.12)	1.99
	＞33.9 s	29.6%	77.6%	1.46 (0.62–3.41)	2.06
ACL‐IgG (GPLU/ml)	＞20	60.0%	73.3%	4.13 (0.58–29.39)	4.02
	＞5.2	57.1%	75.0%	4.00 (0.71–22.43)	3.89
ACL‐IgM (MPLU/ml)	＞12	66.7%	71.9%	5.11 (0.41–63.6)	5.14
	＞1.9	46.2%	77.3%	2.91 (0.66–12.77)	2.97
LA	/	/	/	/	/
Anti‐β2GP1(RU/ml)	＞20	50.0%	69.7%	2.30 (0.13–40.55)	2.55
	＞2.6	62.5%	77.8%	5.83 (1.07–31.76)	6.16

Abbreviations: Anti‐β2GP1, anti‐β2 glycoprotein 1 antibody; ACL, anti cardiolipin antibody; APL, antiphospholipid antibody; APTT, activated partial thromboplastin time; LA, lupus anticoagulant; OR, odds ratio, 95% CI, 95% confidence interval.

### 3.4 Laboratory findings and treatment

The laboratory data for PLT, CRP, ESR, Alb, IgG, fibrinogen, d‐dimers, RF, and anti‐CCP are listed in Table 4. No differences were found between the two groups. The positivity rate of APL was not different between the two groups. The concentration of ACL‐IgG (6.81 ± 2.89 vs. 2.43 ± 0.53, *p* = 0.04) in the case group was significantly higher than that in the control group. In terms of treatment, the number of GC users, initial single daily dose of GC, maximum single daily dose of GC, proportion of hydroxychloroquine used, application of biological agents in initial treatment, and whether the patients were on regular medications or not showed no difference between the two groups.

**Table 4:** Laboratory findings and treatment of patients with or without ONFH.

	ONFH group (*n* = 22)	Control group (*n* = 88)	*p* value
Laboratory findings			
Thrombocytosis (yes/no)	9/13	38/50	1.00
CRP (mg/L)	47.5 ± 7.8	43.9 ± 5.4	0.70
ESR (mm/h)	70.8 ± 8.6	67.5 ± 4.6	0.74
Hypoalbuminemia (yes/no)	3/19	7/81	0.42
Hyperfibrinogenemia (yes/no)	18/4	62/26	0.42
Elevated activated partial thromboplastin time (APTT) (yes/no)	11/11	53/35	0.47
Elevated d‐Dimer (yes/no)	16/2	48/10	0.72
RF positive (yes/no)	14/7	63/18	0.39
Elevated serum IgG (yes/no)	6/13	24/36	0.60
Any APLs positive (yes/no)	2/8	6/19	1.00
ACL‐IgG concentration (GPLU/ml)	6.81 ± 2.89	2.43 ± 0.53	0.04
ACL‐IgM concentration (MPLU/ml)	2.67 ± 2.66	2.73 ± 2.62	0.94
Anti‐β2GP1(RU/ml)	2.27 ± 2.03	2.19 ± 1.57	0.90
Partial APL score	2.97 (2.06, 5.49)	2.06 (0, 4.82)	0.35
Positive of anti‐cyclic citrulline antibody (CCP) (Yes/No)	14/7	40/28	0.67
Treatment before INFH			
NSAIDs (yes/no)	19/3	71/17	0.39
GC (yes/no)	9/13	45/43	0.48
Initial dose of GC (mg)	18.2 ± 16.3	16.3 ± 15.9	0.75
Maximal dose of GC (mg)	24.3 ± 17.6	26.6 ± 19.5	0.74
Hydroxychloroquine (yes/no)	13/9	52/36	1.00
Methotrexate (yes/no)	8/14	37/51	0.41
Biologic therapy at baseline (yes/no)	0/22	6/82	0.60
Antiplatelet drugs (yes/no)	8/14	34/54	0.52
Aspirin (yes/no)	0/22	3/85	0.51
Clopidogrel hydrogen sulfate (yes/no)	8/14	33/55	0.56
Ticagrelor (yes/no)	0/22	1/87	0.80
Dipyridamole (yes/no)	0/22	1/87	0.80
Anticoagulants (yes/no)	9/13	33/55	0.48
Low‐molecular‐weight heparin (yes/no)	8/14	31/57	0.55
Rivaroxaban (yes/no)	1/21	0/88	0.20
Warfarin (yes/no)	0/22	6/82	0.25
Lipid‐lowering drugs (yes/no)	5/17	19/69	1.00
Anti‐osteoporosis drugs (yes/no)	20/2	74/14	0.33
Zoledronic acid (yes/no)	11/11	27/62	0.07
Salmon calcitonin (yes/no)	7/15	24/64	0.43
Calcitriol (yes/no)	12/10	33/55	0.11
Calcium (yes/no)	13/9	54/34	0.52
Drugs for improving blood circulation (yes/no)	15/7	55/33	0.41
Alprostadil (yes/no)	13/9	50/38	0.52
Cilostazol (yes/no)	6/16	17/71	0.29
Regular medication (yes/no)	6/16	42/46	0.10

Abbreviations: ACL‐IgG, anticardiolipin (ACL)‐immunoglobulin G; APL, antiphospholipid antibodies; APTT, activated partial thromboplastin time; CRP, C‐reactive protein; ESR, erythrocyte sedimentation rate; GC, glucocorticoids; NSAID, nonsteroidal antiinflammatory drug; ONFH, osteonecrosis of the femoral head; RF, rheumatoid factor.

### 3.5 Logistic regression analysis

Univariate logistic analysis revealed that hyperlipidemia, SDAI score, DAS28 (ESR), and ACL‐IgG concentrations were risk factors for ONFH in RA. Multivariate logistic regression analysis found SDAI score is the independent risk factor in ONFH patients with RA (Table 5). While, antiphospholipid antibody plays a very small role.

**Table 5:** Multivariate logistic regression analysis of risk factors for ONFH in RA

	Multivariate logistic regression analysis		
	OR	95% CI	*p*
SDAI	1.163	1.013–1.335	**0.03**
DAS28 (ESR)	0.318	0.08–1.263	0.10
Hyperlipidemia	0.003	0.00–0.643	0.54
ACL‐IgG concentration	1.345	0.935–1.933	0.11

Abbreviations: ACL‐IgG, anticardiolipin (ACL)‐immunoglobulin G; ESR, erythrocyte sedimentation rate; SDAI, simplified disease activity index.

## 4 DISCUSSION

The incidence of ONFH in RA is about 5.75%–13.0%.^2^ This study was a retrospective analysis of 683 patients with RA in the past 8 years. Among them, 26 patients developed ONFH, accounting for 3.81%, which was lower than the previously reported incidence. One of the reasons for the low incidence may be that our center only performs pelvic X‐ray or MRI examinations for RA patients with hip discomfort. At the same time, differences in the sample size, diagnostic methods of ONFH, and ethnicity in different studies are also possible reasons for the difference in the incidence.

The pathophysiology of osteonecrosis has not been fully elucidated. Most theories point toward an alteration in the intravascular blood flow as the potential mechanism of ON initiation. Intravascular coagulation can occur as the end result of local vascular impairment. Vascular occlusion occurs because of thrombus formation due to abnormally shaped red blood cells as seen in sickle cells anemia or fat or nitrogen embolism. Extravascular compression may arise secondary to damaged femoral head vessels that permit the accumulation of fat and blood in the extravascular space which leads to alterations in blood flow through local compression. The pathophysiological mechanism arises from an interaction between vascular impairment, altered bone‐cell physiology, risk factors as well as genetics.^9^

We found that hyperlipidemia was a risk factor for ONFH in patients with RA. Studies have shown that hyperlipidemia can damage vascular endothelial cells and weaken the barrier function of the vascular endothelium. It increases the vascular permeability and invasion of mononuclear macrophages.^10^, ^11^ Mononuclear macrophages can transform into foam cells and promote vascular wall atherosclerosis. This results in insufficient blood supply to the bone, leading to bone osteonecrosis.^12^ Hyperlipidemia can also affect the repair and reconstruction of bone tissues. Sticky blood slows the blood flow. Blood in a hypercoagulable state can easily form microthrombi and fat emboli. These can block blood vessels and cause avascular necrosis of bone tissue.^13^, ^14^ Therefore, hyperlipidemia cannot be ignored in clinical diagnosis and treatment.

We also compare the incidence of atheromatosis and coronary atherosclerotic heart disease between two groups. While, we did not find the difference. According to the multivariate logistic regression analysis, we speculate that the origin is not primarily arterial occlusive and antiphospholipid antibody and lipid play a very small role.

A study on the pathology femoral head necrosis in rheumatoid arthritis found that the blood vessels on the synovial surface proliferated, and crawled to the cartilage surface, even eroded the articular cartilage. Finally, the pannus destroyed the articular cartilage and bone. It prompts that the inflammatory component is essential for the formation of osteonecrosis.^15^ In our study, we found that the SDAI and DAS28 (ESR)were higher in RA patients with ONFH. Consistent with our study, high disease activity is closely related to the progression of osteonecrosis.^16^, ^17^ This may be related to chronic inflammation in patients with connective tissue diseases (CTDs). Inflammatory stimulation can damage the vascular endothelium. With disruption of the dynamic balance of coagulation, bleeding or thrombotic events occur.^13^ As for disease activity indicators, we found that the SDAI and DAS28 of the case group were significantly higher than those of the control group. There was no significant difference in objective examination results, including CRP and ESR. I think the inconsistency of these results may be due to the inconsistency of different examiner scores, which is highly biased. This is one of the limitations in our study. Therefore, we must admit that the conclusions of our work are weak. And this is a real‐world study, although the results are somewhat unsatisfactory, the results are true and reliable.

Some studies have reported that patients with SLE demonstrate an association between APL and ONFH. The APL‐S may be an important risk factor for ONFH development. Does APL also have an impact on RA patients? Thirty‐five RA patients underwent testing for APL, and 22.9% of them were positive, which was similar to the previously reported positivity rate.^18^ We did not find a difference in the positivity rate of APL between the two groups. Similarly, the relationship between APL and ONFH has been controversial.^19^, ^20^, ^21^, ^22^ This may be because of the small number of test samples. We included RA patients with hip discomfort in the study, and we missed nonsymptomatic osteonecrosis patients, which are relatively frequent and could be significant subclinical patients who explains this mild difference. Some studies have reported that APL can increase the probability of ONFH. The APL‐S in the RA with ONFH group was similar in the group without ONFH. The APL‐S may be of great significance in predicting the risk of ONFH in SLE^23^ but not in RA. In this study, we tried our best effort to explore the relationship between APLs and ONFH, including ACL‐IgM, ACL‐IgG, Anti‐β2GP1, even partial APL score. We indeed found that the concentration of ACL‐IgG (6.81 ± 2.89 vs. 2.43 ± 0.53, *p* = 0.04) in the case group was significantly higher than that in the control group. But the concentration of ACL‐IgG was still in the normal ranges (ACL IgG＜12.0 RU/ml). As far as we know, no research about the correlation between rheumatoid arthritis femoral head necrosis and APLs has been found. But in SLE, the relationship between APLs and osteonecrosis has been widely explored. We performed a meta‐analysis and included a total of 22 articles and 3054 patients. The following conclusions were found: the positivities of anticardiolipin antibody (ACL) (odds ratio [OR] = 0.87, 95% confidence interval [CI] [0.64, 1.18], *p* = 0.37), IgG ACL (OR = 1.39, 95% CI [0.68, 2.83], *p* = 0.37), IgM ACL (OR = 2.07, 95% CI [0.88, 4.90], *p* = 0.09), LA (OR = 1.34, 95% CI [0.69, 2.60], *p* = 0.39), and APS (OR = 1.074, 95% CI [0.74, 1.55], *p* = 0.70) in SLE is not associated with ON. Two articles explored the relationship between β2GP1 antibody and ON. One study showed that IgG or IgM β2GP1 had no association with ON. The other one found the relationship between β2GP1 antibody and ON. While it did not provide the type of antibody. So, SLE patients also demonstrated a weak association between APLs and osteonecrosis. So, we speculate that it may have little effect on femoral head necrosis.

Some reports found necrosis of chondrocytes and cancellous bone in RA patients. An increase of osteoblastic and/or osteocytic apoptosis in the femoral head might be one of the main features of the pathogenesis of ON. A study found the evidence of osteoporosis in the femoral head bone in RA patients. This also confirms that osteoporosis is the pathophysiological process of femoral head necrosis. In addition, although we did not find a significant difference in the incidence of osteoporosis and the use of zoledronic acid between the two groups, almost 50% of patients with ONFH used zoledronic acid against 30% in the matched, this is biologically a better explanation for the occurrence of osteonecrosis. In our study, we found that high disease activity is a risk factor for the development of necrosis of the femoral head, and we speculate that the origin is not primarily arterial occlusive, and the inflammatory component is essential. As we all know, GC are a known risk factor for osteonecrosis. Naturally, we can make an inference that in more active patients, the use of GC is higher, as a result, the probability of necrosis of the femoral head will increase. While, with the rapid development and application of biological agents, RA patients with higher disease activity may not necessarily use higher doses of GC, and the wide application of biological agents has reduced the dosage of GC greatly. We can see from Table 4, nobody uses biological agents in ONFH group, this is the potential cause of femoral head necrosis.

Many studies on SLE and nephrotic syndrome (NS) have reported that long‐term use of GC is a risk factor for ONFH.^24^, ^25^ GC can reduce the viability of osteoblasts and hinder bone matrix formation. GC can increase the apoptotic process of bone cells, result in osteoporosis, and ultimately lead to osteonecrosis.^13^ However, in a 10‐year follow‐up of patients with inflammatory arthritis, Colwell et al. did not find a direct correlation between the dose of GC and ONFH.^26^ Our study analyzed the effects of the initial and maximum doses of GC on the occurrence of ONFH, but no particular effects were found. It does not mean that GC are not the cause of femoral head necrosis in rheumatoid arthritis, because in addition to the initial dose and maximum dose, regular treatment or not, duration of GC use, and total amount of use is also closely related to. In our study, 56% of patients did not take medication regularly, so it is impossible to trace the duration and total amount of GC use.

This study analyzed the risk factors for ONFH in patients with RA in a single center over a period of 8 years. The longest follow‐up time was 20.5 years, which was in a real‐world study. This study has the following limitations: (1) it was a retrospective observational study. (2) Not all patients underwent APL testing. (3) The sample size was small and needed to be further expanded. (4) CDAI, SDAI, and DAS28 (ESR) are variables with very high oscillation dependent on the examiner to be easily biased.

In summary, we found that hyperlipidemia, the SDAI score, the CDAI score, and ACL‐IgG concentrations are risk factors for ONFH in patients with RA. The pathogenesis is related to many factors. While antiphospholipid antibody is not the key factor. The results of studies about ONFH in patients with RA were inconsistent. Nonsymptomatic osteonecrosis patients could be significant subclinical patients who explain this question. And the risk factors for ONFH in RA need to be investigated further in more studies.

## AUTHOR CONTRIBUTIONS

Wei Qijiao collected and analyzed clinical data and drafted the article. Chen Zhihan designed this topic and approved the final version of manuscript. Lin He revised the manuscript carefully. Lin Diantian, Yan Qing and Gao Fei helped Wei Qijiao to collect and interpret data for the work.

## CONFLICTS OF INTEREST

The authors declare no conflicts of interest.

## Data Availability

No additional data are available.
